# A Note on the Eigensystem of the Covariance Matrix of Dichotomous Guttman Items

**DOI:** 10.3389/fpsyg.2015.01767

**Published:** 2015-12-01

**Authors:** Clintin P. Davis-Stober, Jean-Paul Doignon, Reinhard Suck

**Affiliations:** ^1^Department of Psychological Sciences, University of MissouriColumbia, MO, USA; ^2^Department of Mathematics, Université Libre de BruxellesBrussels, Belgium; ^3^Universität OsnabrückOsnabrück, Germany

**Keywords:** Guttman scale, dichotomous items, Rasch model, principal component analysis, eigenvalues, eigenvectors

## Abstract

We consider the covariance matrix for dichotomous Guttman items under a set of uniformity conditions, and obtain closed-form expressions for the eigenvalues and eigenvectors of the matrix. In particular, we describe the eigenvalues and eigenvectors of the matrix in terms of trigonometric functions of the number of items. Our results parallel those of Zwick (1987) for the correlation matrix under the same uniformity conditions. We provide an explanation for certain properties of principal components under Guttman scalability which have been first reported by Guttman (1950).

## 1. Introduction

Guttman scales form the conceptual foundation for modern Item Response Theory (IRT). For example, Guttman scales underlie the Rasch model (e.g., Andrich, 1985) as well as Mokken scales (e.g., van Schuur, 2003),—see Tenenhaus and Young (1985) and Lord and Novick (1968) for classic reviews and discussions of Guttman scaling. Under the auspices of understanding the principal component structure of unidimensional scales, Guttman (1950) derived several important properties relating to the correlation matrix of perfect dichotomous Guttman items. Later work by Zwick (1987) identified that the eigenvalues corresponding to this matrix can be written as simple functions of the number of items, under a set of uniformity conditions.

In this brief note, we extend the results of Zwick (1987) by considering the covariance matrix of dichotomous Guttman items under these same uniformity conditions. We derive closed-form solutions for the eigenvalues and eigenvectors of this matrix, for any number of items. In particular, we provide expressions in terms of simple trigonometric functions of the number of items. These expressions lead to a simple explanation of the signing relationships among principal components for Guttman scales first described by Guttman (1950).

## 2. Main results

The core idea of a Guttman scale is that the set of items under consideration forms a unidimensional scale, i.e., if a person obtains a correct response to an item then this person would obtain a correct response to all “easier” items. Table 1 presents a matrix of response patterns conforming to a perfect Guttman scale for five items, with Item 5 being the most “difficult” and Item 1 being the “easiest.”

**Table 1:** **An example of a perfect Guttman scale for five items**.

**Response pattern**	**Item 1**	**Item 2**	**Item 3**	**Item 4**	**Item 5**
1	0	0	0	0	0
2	1	0	0	0	0
3	1	1	0	0	0
4	1	1	1	0	0
5	1	1	1	1	0
6	1	1	1	1	1

*A value of 0 indicates an incorrect response to the item, a value of 1 indicates a correct response*.

As in Zwick (1987), we consider the following two assumptions. First, we assume that all items are distinct, i.e., no two items produce identical responses for all possible response patterns. Second, we assume that the probability of obtaining each response pattern is $\frac{1}{n+1}$, where *n* is the number of items, i.e., a uniform distribution over response patterns. This last assumption is rather strong, given that responses are typically modeled using a normal distribution. While we assume uniformly distributed response patterns primarily for mathematical tractability, we demonstrate later via simulations that our results approximate those obtained from a normal distribution under highly discriminating items that are equally spaced by difficulty.

Under our assumptions, the covariance between any items *i* and *j*, with *i* ≤ *j*, is equal to the following:

(1) $$\begin{array}{r} \sigma_{i,j}^{cov}=\frac{i\left(n+1-j\right)}{{\left(n+1\right)}^{2}},\;\forall i,j\in \{1,2,\ldots n\}\;\text{with}\;i\leq j. \end{array}$$

As one would expect, Equation (1) is closely related to the Pearson product-moment correlation, which, as described by Zwick (1987), is equal to:

(2) $$\begin{array}{r} Corr\left(i,j\right)=\sqrt{\frac{i\left(n+1-j\right)}{j\left(n+1-i\right)}},\;\forall i,j\in \{1,2,\ldots n\}\;\text{with}\;i\leq j. \end{array}$$

Parallel to Zwick (1987) and Guttman (1950), who handled the correlation matrix, we consider the *n* × *n* covariance matrix defined by Equation (1). We first provide the *n* distinct eigenvalues.

**Proposition** 1. *The covariance matrix σ^cov^, with entries given by Equation* (1), *has its eigenvalues equal to (in decreasing order)*

(3) $$\lambda_{i}^{cov}=\frac{1}{\left(n+1)(2-2\cos(\frac{i\pi }{n+1})\right)},\;i=1,2,\ldots ,n.$$

The proof is in the Appendix.

Note how *i* and *n* determine the period of the cosine term in the denominator of the right-hand side of Equation (3). From the same equation, the maximal eigenvalue for any fixed number of items *n* is equal to $\lambda_{n}^{max}=\frac{1}{\left(n+1\right)\left(2\;-\;2\cos\left(\frac{\pi }{n+1}\right)\right)}$. Note that as *n* → ∞, $\lambda_{n}^{max}\to \infty$. Also, the eigenvalues of the covariance matrix are very different from the eigenvalues of the Pearson product correlation matrix, which, as described by Zwick (1987), are equal to $\lambda_{i}^{corr}=\frac{n+1}{i\left(i+1\right)}$.

The eigenvectors of the covariance matrix also have an elegant, closed-form expression.

**Proposition** 2. *For the covariance matrix σ^*cov*^ defined by Equation* (1), *an eigenvector *P*_*i*_ of eigenvalue $\lambda_{i}^{cov}$, with *i* =* 1, *2, …, *n* (as in Proposition 1), results from setting*

(4) $$\begin{array}{r} P_{i,m}=\sin\left(\frac{im\pi }{n+1}\right),\;m=1,2,\ldots ,n. \end{array}$$

The proof is in the Appendix.

Guttman (1950) derived a series of relationships on the eigenvector components of correlation matrices based on perfect, “error free” scales. Let *sgn*(*x*) be the sign function of the value *x*. Define a *sign change* of an eigenvector *P*_*i, m*_ as a value *j* such that *sgn*(*P*_*i, j*_) ≠ *sgn*(*P*_*i, j*+1_), *j* ∈ {1, 2, …, *n*}. As described in Guttman (1950), for *n*-many items there exists exactly one eigenvector with no sign changes, one eigenvector with a single sign change, one with two sign changes, and so on, with the eigenvector corresponding to the smallest eigenvalue having exactly *n*–1 sign changes. This symmetry can be seen in Table 2, which presents the eigenvectors in Equation (4) for *n* = 5. As made explicit by Equation (4), these sign changes result from the symmetry of the sine function as the values of *i* and *m* vary.

**Table 2:** **This table presents the eigenvector components of the covariance matrix for ***n*** = 5**.

***P*_1_**	***P*_2_**	***P*_3_**	***P*_4_**	***P*_5_**
$\frac{1}{2}$	$\frac{\sqrt{3}}{2}$	1	$\frac{\sqrt{3}}{2}$	$\frac{1}{2}$
$\frac{\sqrt{3}}{2}$	$\frac{\sqrt{3}}{2}$	0	$-\frac{\sqrt{3}}{2}$	$-\frac{\sqrt{3}}{2}$
1	0	–1	0	1
$\frac{\sqrt{3}}{2}$	$-\frac{\sqrt{3}}{2}$	0	$\frac{\sqrt{3}}{2}$	$-\frac{\sqrt{3}}{2}$
$\frac{1}{2}$	$-\frac{\sqrt{3}}{2}$	1	$-\frac{\sqrt{3}}{2}$	$\frac{1}{2}$

*The eigenvectors are arrayed in descending order according to the corresponding eigenvalue, i.e., P_1_ is the eigenvector corresponding to the maximal eigenvalue*.

## 3. Comparison to IRT data

In this section, we illustrate how our analytic results could be used to evaluate responses conforming to modern IRT models. We consider the well-known *two parameter logistic* (2PL) model, where the probability of a correct response to item *i* is defined as follows:

(5) $$\begin{array}{r} p_{i}\left(\theta \right)=\frac{1}{1+\exp^{-a_{i}\left(\theta -b_{i}\right)}}, \end{array}$$

where $a_{i}\in {\mathbb{R}}^{+}$ is the item discrimination parameter, *b*_*i*_ ∈ ℝ is the item difficulty parameter and θ ∈ ℝ is the person-specific ability parameter.

From the perspective of the 2PL model, Guttman items are obtained by letting the *a*_*i*_ (item discrimination) parameter values become arbitrarily large (e.g., van Schuur, 2003), i.e., the probability of a test taker correctly answering an item given that their latent skill is higher (lower, resp.) than the item difficulty is 1 (0 resp.). Our results provide a new perspective on the item covariance and principal component structure of 2PL items under the idealized conditions of a Guttman scale. Indeed, one could consider the eigenvalues and eigenvectors in Equations (3–4) as an error-free ideal for such response data, under our assumption of a uniform distribution over response patterns.

In the next section, we compare our results to simulated data that relax the assumption of a uniform distribution over response patterns. In the first simulation study, we compare our results to data generated from a Rasch model (Equation 5 with *a*_*i*_ = 1, *i* = 1, 2, …, *n*) where the person specific ability parameter, θ, is randomly drawn from a standard normal distribution. For the second simulation study, we consider a setup nearly identical to the first, with the exception that we consider large values of *a*_*i*_ for each item, i.e., high discrimination among items.

### 3.1. Simulation study 1

For this study, we considered six conditions comprised of: 4, 6, 8, 16, 32, and 64 test items. For each condition, the difficulty of the items, *b*_*i*_, was equally spaced along the interval [−1, 1]. For each condition, we randomly sampled 5000 values of θ from a standard normal distribution (e.g., Anderson et al., 2007). We obtained simulated responses to the items by applying the sampled θ values, and item difficulties, *b*_*i*_, to Equation (5), with *a*_*i*_ = 1 for all test items, i.e., a Rasch model. Thus, for each condition, we have 5000 simulated responses to the test items.

**Figure 1:**
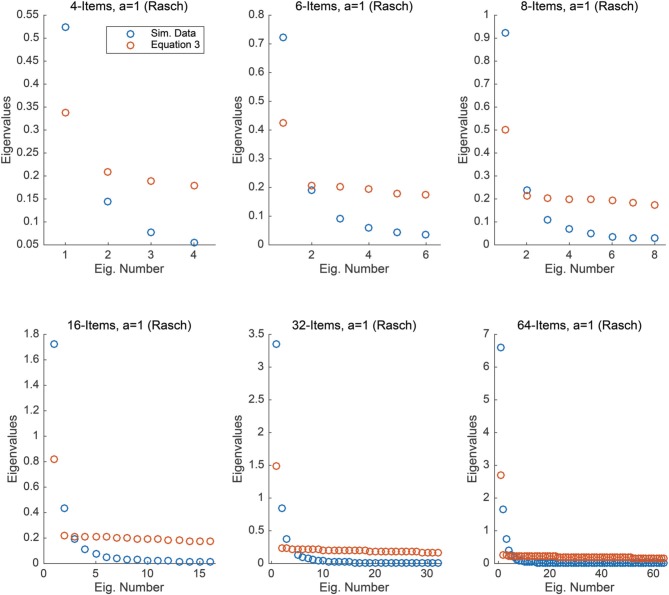
**Each plot compares the eigenvalues obtained from Equation (3) to those obtained from simulated Rasch data under the assumption that θ ~ *N*(0, 1) under *n* = 4, 6, 8, 16, 32, and 64 items**.

For each condition, we computed the covariance matrix of the items using the 5000 simulated responses, i.e., we calculated the sample covariance of the 5000 responses. We then numerically calculated the eigenvalues of this covariance matrix. Figure 1 compares the eigenvalues obtained from the simulated data to the eigenvalues obtained from Equation (3), for each condition. It is interesting to note that the largest eigenvalue for the simulated data is always larger than the maximal eigenvalue obtained via Equation (3), this is similar to results obtained by Zwick (1987) within the context of the Guttman correlation matrix. In general, moving to a probabilistic response model (the Rasch model) and sampling the θ values from a normal distribution appears to yield covariance eigenvalues that greatly differ from those obtained in Equation (3). As we show in the next study, improving item discrimination will yield different results.

### 3.2. Simulation study 2

In this simulation study, we consider nearly identical conditions to the first, with the exception that the item discrimination parameters, *a*_*i*_, are large in size, indicating excellent item discrimination. As in the previous study, we considered six conditions comprised of: 4, 6, 8, 16, 32, and 64 test items. For each condition, the difficulty of the items, *b*_*i*_, was equally spaced along the interval [−1, 1]. As before, for each condition, we randomly sampled 5000 values of θ from a standard normal distribution. We obtained simulated responses to the items by applying the sampled θ values, and item difficulties, *b*_*i*_, to Equation (5), with *a*_*i*_ = 3, *i* = 1, 2, …, *n*, indicating excellent item discrimination. As before, for each condition, we have 5000 simulated responses to the test items.

**Figure 2:**
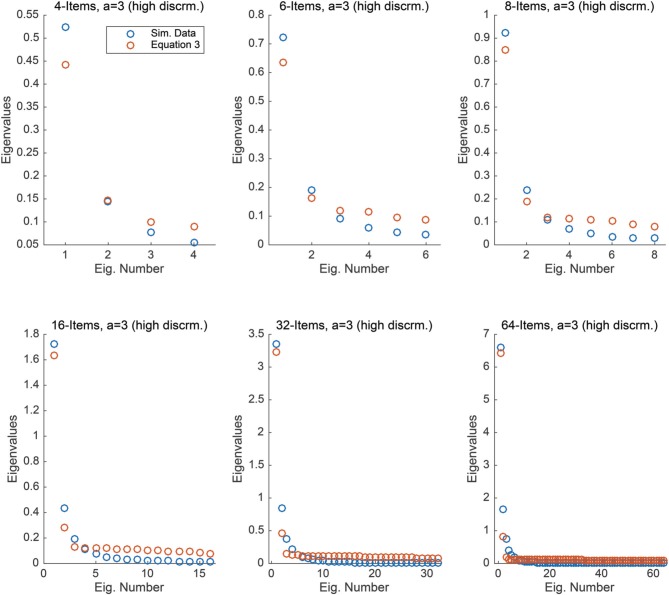
**Each plot compares the eigenvalues obtained from Equation (3) to those obtained from simulated 2PL data with high item discrimination under the assumption that θ ~ *N*(0, 1) under *n* = 4, 6, 8, 16, 32, and 64 items**.

For each condition, we computed the covariance matrix of the 5000 simulated responses and numerically calculated the eigenvalues of the generated covariance matrix for each condition. Figure 2 compares the eigenvalues from these simulated data to the eigenvalues obtained via Equation (3), for each condition. It is interesting to note that there is a much closer correspondence between the two sets of eigenvalues under these conditions. Further, this relationship becomes stronger as the number of equally spaced items increases, yielding nearly a perfect match to the maximal eigenvalue as the number of items reaches 32 and 64.

This study illustrates that our analytic results, which are derived under the strong assumption of uniformly distributed response patterns, may be useful as an approximation even when the ability parameter is normally distributed. This approximation is best when the difficulty range of the items are within a single standard deviation of the mean and the items have excellent discriminability. As the range of the item difficulty increases and/or the variance of the ability parameter distribution shrinks, the approximation becomes much poorer. Our Matlab code for generating these graphs and exploring other configurations is available as an online supplement.

## 4. Conclusion

We derived closed-form solutions for the eigenvalues and eigenvectors of the covariance matrix of dichotomous Guttman items, under a uniform sampling assumption. We demonstrated that these eigenvalues and eigenvectors are simple trigonometric functions of the number of items, *n*. Our results parallel those of Zwick (1987), who examined the eigenvalues of the correlation matrix of dichotomous Guttman items under the same uniformity assumptions. It remains an open question whether the eigenvectors of the correlation matrix, as investigated by Zwick (1987), can also be solved for explicitly.

### Conflict of interest statement

The authors declare that the research was conducted in the absence of any commercial or financial relationships that could be construed as a potential conflict of interest.

## Appendix

### A. Proofs of propositions 1 and 2

To prove the main results of the paper, we first derive the general inverse of the covariance matrix of Guttman items under our uniformity assumptions. This inverse has a special tridiagonal form. From this tridiagonal form, we apply known algebraic results to obtain the required eigenvalues and eigenvectors.

Define *X* as the (*n* + 1) × *n* matrix of perfect Guttman scores under the specified uniformity assumptions. The rows of this matrix correspond to response patterns while the columns correspond to Guttman items, see also Table 1. This matrix has zeros on the diagonal and above, and all elements below the diagonal are ones:

$$X=\left(\begin{array}{ccccc} 0 & 0 & 0 & \ldots & 0\\ 1 & 0 & 0 & \ldots & 0\\ 1 & 1 & 0 & \ldots & 0\\ \vdots & \vdots & \vdots & \ddots & \vdots \\ 1 & 1 & 1 & \ldots & 1 \end{array}\right).$$

We denote by *e*^(*i*)^ the *i*-th vector of the canonical basis of ℝ^*n* + 1^, and by *v*^(*i*)^ the *i*-th column vector of *X*. For later use, it is convenient to introduce also *v*^(0)^ = (1, 1, …, 1)′ and *v*^(*n* + 1)^ = (0, 0, …, 0)′. Thus in ℝ^*n* + 1^ we have for *i* = 1, 2, …, *n* + 1,

$$v^{\left(i\right)}=v^{\left(i-1\right)}-e^{\left(i\right)},$$

and then also, for *i* = 1, 2, …, *n*,

(A1) $$\begin{array}{l} v^{\left(i\right)}\text{ = }\frac{1}{2}\left(v^{\left(i-1\right)}-e^{\left(i\right)}\right)+\frac{1}{2}\left(v^{\left(i+1\right)}+e^{\left(i+1\right)}\right)\\ \text{ = }\frac{1}{2}\left(v^{\left(i-1\right)}+v^{\left(i+1\right)}\right)+\frac{1}{2}\left(e^{\left(i+1\right)}-e^{\left(i\right)}\right). \end{array}$$

Obtaining the eigenvalues and eigenvectors of the covariance matrix via the columns *v*^(*i*)^ of *X* can be done in five steps:

1. centering each vector *v*^(*i*)^ (for *i* = 1, 2, …, *n*), that is, subtracting the mean of all components of *v*^(*i*)^ from each component; let us denote by ṽ^(*i*)^ the resulting vector;
2. computing the element *S*_*ij*_ of the matrix *S* as the scalar product ṽ^(*i*)^ · ṽ^(*j*)^;
3. deriving the inverse of *S* by taking into account a special property of the rows of *S* (see below);
4. inferring the eigenvalues and eigenvectors of *S*^−1^ (then also of *S*) from the special form of *S*^−1^, a tridiagonal matrix;
5. finally, observing that the covariance matrix equals $\sigma^{cov}=\frac{1}{\left(n+1\right)}S$, thus obtaining the eigenvalues and eigenvectors of ${\hat{\sigma }}^{cov}$.

Let us rephrase these steps in a more geometric fashion. In Step 1, ṽ^(*i*)^ is the image of *v*^(*i*)^ by the orthogonal projection from ℝ^*n* + 1^ to the hyperplane *H* with equation $\overset{n+1}{\underset{i=1}{\sum }}x_{i}=0$ (indeed, ṽ^(*i*)^ ∈ *H* and furthermore ṽ^(*i*)^ − *v*^(*i*)^, a constant vector, is orthogonal to *H*). Moreover, notice that *e*^(*i*)^ − *e*^(*j*)^ belongs to *H* and so projects onto itself. Consequently, for *i* = 1, 2, …, *n*, we derive from Equation (A1)

(A2) $$\begin{array}{l} {ṽ}^{\left(i\right)}=\frac{1}{2}\left({ṽ}^{\left(i-1\right)}+{ṽ}^{\left(i+1\right)}\right)+\frac{1}{2}\left(e^{\left(i+1\right)}-e^{\left(i\right)}\right). \end{array}$$

In Step 2, we compute *S*_*i, j*_ as the scalar product of ṽ^(*i*)^ with ṽ^(*j*)^. Taking the scalar product of both sides of the previous equation with ṽ^(*j*)^, we get for *i* = 2, 3, …, *n* − 1 and *j* = 1, 2, …, *n*

$$S_{i,j}=\frac{1}{2}\left(S_{i-1,j}+S_{i+1,j}\right)+\frac{1}{2}\left({ṽ}_{i+1}^{\left(j\right)}-{ṽ}_{i}^{\left(j\right)}\right).$$

Now because

$${\tilde{v}}_{i+1}^{\left(j\right)}-{\tilde{v}}_{i}^{\left(j\right)}=v_{i+1}^{\left(j\right)}-v_{i}^{\left(j\right)}=\{\begin{array}{ll} 1 & \text{if }i=j,\\ 0 & \text{otherwise}, \end{array}$$

we see that row *S*_*i*, •_ is the mean of rows *S*_*i* − 1, •_ and *S*_*i* + 1, •_ except for its diagonal element which is $\frac{1}{2}$ more than the mean. This holds for *i* = 2, 3, …, *n* − 1. By considering extraneous rows *S*_0, •_ = (0, 0, …, 0) and *S*_*n* + 1, •_ = (0, 0, …, 0), we can also allow *i* = 1 and *i* = *n* [this follows again from (A2), considered now for *i* = 1 and *i* = *n*, together with ṽ^(0)^ = ṽ^(*n* + 1)^ = (0, 0, …, 0)′]. This special property of *S* immediately translates into the following expression for the inverse matrix of *S*:

$$S^{-1}=\left(\begin{array}{ccccccc} 2 & -1 & 0 & 0 & 0 & \ldots & 0\\ -1 & 2 & -1 & 0 & 0 & \ldots & 0\\ 0 & -1 & 2 & -1 & 0 & \ldots & 0\\ 0 & 0 & -1 & 2 & -1 & \ldots & 0\\ \vdots & \vdots & \vdots & \ddots & \ddots & \ddots & \vdots \\ 0 & 0 & \ldots & 0 & -1 & 2 & -1\\ 0 & 0 & \ldots & 0 & 0 & -1 & 2 \end{array}\right).$$

(indeed, the product of the above matrix with *S* equals the identity matrix).

The form of *S*^−1^ follows a particular tridiagonal form that has been extensively studied in the mathematics literature. Elliott (1953) and Gregory and Karney (1969) identified that the eigenvalues λ_*i*_ of *S*^−1^, and (selected) corresponding eigenvectors *P*_*i*_, for *i* = 1, 2, …, *n*, are given by $\lambda_{i}=2-2\cos\left(\frac{i\pi }{n+1}\right)$ (in increasing order) and $P_{i,m}=\sin\left(\frac{im\pi }{n+1}\right)$ respectively (where *m* = 1, 2, …, *n*). These results were later extended to more general tridiagonal matrices by Yueh (2005), see also Yueh and Cheng (2008) and Bünger (2014).

Because the covariance matrix σ^*cov*^ equals $\frac{1}{\left(n+1\right)}S$, the eigenvalues of σ^*cov*^ are equal to $\frac{1}{n+1}$ times those of *S*, so they are also $\frac{1}{n+1}$ times the inverses of the eigenvalues of *S*^−1^.

Proposition 1 now follows from the fact that the eigenvalues of σ^*cov*^ are equal to $\frac{1}{n+1}$ times the reciprocals of the eigenvalues of *S*^−1^, and Proposition 2 from the fact the matrices σ^*cov*^, *S*, and *S*^−1^ have the same eigenvectors. This completes the proof.     □

## References

[B1] Anderson C. J. Li Z. Vermunt J. K. (2007). Estimation of models in a Rasch family for polytomous items and multiple latent variables. J. Stat Soft. 20, 1–36. 10.18637/jss.v020.i06

[B2] Andrich D. (1985). An elaboration of Guttman scaling with Rasch models for measurement, in Sociological Methodology 1985. Jossey-Bass Social and Behavioral Science Series, ed Tuma N. B. (San Francisco, CA: Jossey-Bass), 33–80.

[B3] Bünger F. (2014). Inverses, determinants, eigenvalues, and eigenvectors of real symmetric Toeplitz matrices with linearly increasing entries. Linear Algebra Appl. 459, 595–619. 10.1016/j.laa.2014.07.023

[B4] Elliott J. F. (1953). The Characteristic Roots of Certain Real Symmetric Matrices. Masters thesis, University of Tennessee.

[B5] Gregory R. T. Karney D. (1969). A Collection of Matrices for Testing Computational Algorithm. New York, NY: Wiley-Interscience.

[B6] Guttman L. (1950). The principal components of scale analysis, in Measurement and Prediction, eds Stouffer S. A. Guttman L. Suchman E. A. Lazarsfeld P. F. Star S. A. Clausen J. A. (Princeton, NJ: Princeton University Press), 312–361.

[B7] Lord F. M. Novick M. (1968). Statistical Theories of Mental Test Scores. Reading, MA: Addison-Wesley.

[B8] Tenenhaus M. Young F. W. (1985). An analysis and synthesis of multiple correspondence analysis, optimal scaling, dual scaling, homogeneity analysis and other methods for quantifying categorical multivariate data. Psychometrika 50, 91–119. 10.1007/BF02294151

[B9] van Schuur W. H. (2003). Mokken scale analysis: between the Guttman scale and parametric item response theory. Polit. Anal. 11, 139–163. 10.1093/pan/mpg002

[B10] Yueh W.-C. (2005). Eigenvalues of several tridiagonal matrices. Appl. Math. E-Notes 5, 66–74.

[B11] Yueh W.-C. Cheng S. S. (2008). Explicit eigenvalues and inverses of tridiagonal Toeplitz matrices with four perturbed corners. ANZIAM J. 49, 361–387. 10.1017/S1446181108000102

[B12] Zwick R. (1987). Some properties of the correlation matrix of dichotomous Guttman items. Psychometrika 52, 515–520. 10.1007/BF02294816

